# A Tet-Off gene expression system for validation of antifungal drug targets in a murine invasive pulmonary aspergillosis model

**DOI:** 10.1038/s41598-017-18868-9

**Published:** 2018-01-11

**Authors:** Yutian Peng, Hua Zhang, Min Xu, Man-Wah Tan

**Affiliations:** 1 Infectious Diseases Department, Genentech Inc., South San Francisco, California, 94080 USA; 2 Translational Immunology Department, Genentech Inc., South San Francisco, California, 94080 USA

## Abstract

*Aspergillus fumigatus* is one of the major causes of invasive pulmonary aspergillosis in immunocompromised patients. Novel antifungal therapy is in urgent need due to emerging resistance and adverse toxicity of current antifungal drugs. Gene products that are essential for *Aspergillus* viability during infection are attractive drug targets. To characterize these genes *in vivo* we developed a Tet-Off gene expression system in *A. fumigatus*, whereby the administration of doxycycline resulted in down regulation of the gene whose expression is under the control of the Tet-Off promoter. We tested the system on two potential drug targets, inosine 5′-monophosphate dehydrogenase (*IMPDH*) and L-ornithine N^5^-oxygenase (*sidA*) in a murine invasive pulmonary aspergillosis model. We show that depletion of *IMPDH* attenuated but did not completely abolish virulence *in vivo* whereas turning off the expression of *sidA*, which is required for iron acquisition, resulted in avirulence. We also investigated whether *sidA* expression could be controlled in a time-dependent manner in mice. Our results demonstrated that timing of doxycycline administration dramatically affects survival rate, suggesting that this genetic system can be used for testing whether an antifungal drug target is critical for fungal growth post-infection.

## Introduction

*Aspergillus fumigatus* is one of the most common filamentous fungi associated with severe invasive infections. Despite the availability of four major classes of anti-fungal therapies – polyenes, pyrimidine analogs, echinocandins and triazoles – invasive pulmonary aspergillosis still results in high mortality rate in immunocompromised patients. Polyenes, such as amphotericin B, bind to egosterol and destabilize cell membrane. Toxicity of amphotericin B has been reduced by using liposome formulation. Pyrimidine analogs, such as 5-fluorocytosine, inhibits pyrimidine biosynthesis. Echinocandins inhibit the β-1, 3 glucan synthase and therefore block cell wall biosynthesis. Triazoles inhibit specifically egosterol biosynthesis. Due to the side effects of these drugs and rising resistance, more efficacious antifungal drugs with novel therapeutic modalities or mechanisms of action are urgently needed^1^.

Genes essential for *A. fumigatus* viability or pathogenesis serve as potential drug targets. Conditional gene expression is a central strategy for characterizing the functions of essential genes. In addition, conditional gene expression systems allow for the level of a target gene to be regulated in a dose-dependent and/or in a time-dependent manner, which is important for discovery of gene function in physiologically relevant conditions.

Several conditional gene expression systems have been developed for *A. fumigatus*, including regulation by the *Aspergillus nidulans alcA* promoter^2^, *A. fumigatus NiiA* promoter^3^, and *Escherichia coli* tetracycline-controlled promoter^4^. *A. nidulans alcA* gene encodes alcohol dehydrogenase I, whose expression can be induced by ethanol or threonine and repressed by the presence of glucose. Tight regulation is achievable *in vitro*, however, controlling the activity of the *alcA* promoter is not possible in animal models. Recently, a nitrogen-regulated *A. fumigatus NiiA* promoter was used to identify essential genes in a large-scale functional study^3^. The *NiiA* promoter can be turned on in the absence of ammonium and the presence of nitrate, and turned off in the presence of ammonium regardless of the other nitrogen. As mouse serum and tissues naturally contain ammonium, expression of a given *NiiA* promoter driven gene can be sufficiently repressed *in vivo* ^3^. Similar to the *alcA* promoter, the drawback of the *NiiA* promoter is the inability to turn on/off a target gene in an animal model once infection is established because controlling the level of nitrate and ammonium in the mouse is not trivial. By contrast, the tetracycline-controlled transcription activation system provides an attractive opportunity to use non-native molecules such as tetracycline or doxycycline to regulate fungal gene expression. Indeed, the Tet-On and Tet-Off system has been successfully adopted in various eukaryotic cells. In the Tet-Off system, tetracycline-controlled transactivator (tTA) activates transcription by binding to the *tet* operator sequences (*tetO*) in the absence of tetracycline or doxycycline. Addition of tetracycline or doxycycline prevents tTA from binding *tetO* and therefore blocks transcription. Conversely, in the Tet-On system, a ‘reverse’ tetracycline transactivator (rtTA) only binds to *tetO* in the presence of tetracycline or doxycycline^5,6^. The Tet-On and Tet-Off system was first introduced in *A*. *fumigatus* on two separate plasmids, which contain *tetO* and tTA/rtTA, respectively^4^. However, the leakiness of the promoter compromises the reliability of phenotypic analyses^7^. More recently, the Tet-On system was improved and upgraded to one module that can be integrated to *A. fumigatus* genome^7^. A following study validated this Tet-On system in both murine pulmonary and systemic infection of *A. fumigatus*. Under the Tet-On promoter, genes required for aromatic amino acid biosynthesis were turned on by supplementing drinking water with doxycycline, resulting in infection of *A. fumigatus* ^8,9^. Subsequently, a Tet-Off gene expression system was created by substituting rtTA2S-M2 with a tTA2 transactivator in the Tet-On cassette described above. Under this system, gene expression tested was effectively inhibited in the presence of doxycycline in vitro^10^.

In this study, we created a Tet-Off gene expression system in *A. fumigatus* and used it to investigate whether two potential target genes are essential for virulence of *A. fumigatus* in a murine invasive pulmonary aspergillosis model. We further tested the utility of this system to repress target gene expression *in vivo* in a time-dependent manner.

## Results and Discussion

### *IMPDH* is conditionally essential for growth of *A. fumigatus in vitro*

**Figure 1:**
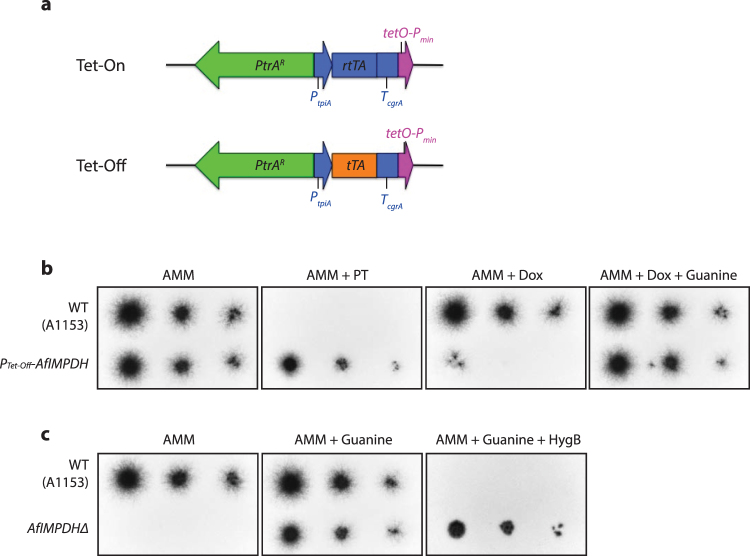
*AfIMPDH* is conditionally essential for *A. fumigatus in vitro*. (**a**) Schematic representation of the Tet-Off cassette. The Tet-Off cassette is composed of the pyrithiamine resistance cassette (*PtrA* ^*R*^), the *tpiA* promoter of *A. nidulans* (*P*_*tpiA*_)^33^, the tetracycline transactivator *tTA2* ^*S*^ (Clontech), the terminating region of *cgrA* from *A. fumigatus* (*T*_*cgrA*_)^34^, and the chimeric *tetO-P*_*min*_ promoter. (**b**) Doxycycline-dependent growth of the *P*_*Tet-Off*_ *-AfIMPDH* strain. Approximate 500, 50 and 5 conidia of wild type (A1151) and the *P*_*Tet-Off*_ *-AfIMPDH* strain were spotted on the AMM agar plates supplemented with the indicated compounds. PT: pyrithiamine, 0.5 μg/mL; Dox: doxycycline, 1 μg/mL; Guanine: 500 μM, HygB: hygromycin B, 200 μg/mL. The plates were subsequently incubated at 37 °C for 36–48 hours and imaged. (**c**) Growth of the *AfIMPDHΔ* strain. Conidia of the wild type and the mutant strains were spotted and grown as described in (**b**).

To construct a Tet-Off expression system for *A. fumigatus*, we modified a previously published Tet-On expression cassette^7^. We replaced the reverse tetracycline transactivator *rtTA2* ^*S*^ *-M2* with a tetracycline transactivator tTA-Advanced (*tTA2* ^S^) which was optimized for tight binding to *tetO* during induction (Clontech) (Fig. 1a). To test whether our Tet-Off cassette can effectively turn off expression of a given target gene, we integrated this Tet-Off cassette at the promoter region of *A. fumigatus* inosine 5′-monophosphate dehydrogenase (*AfIMPDH*) by homologous recombination in a non-homologous end-joining-deficient strain^11^.

IMPDH catalyzes the first step in *de novo* guanine biosynthesis by converting inosine 5'-monophosphate (IMP) to xanthosine 5′-monophosphate (XMP). A specific inhibitor to human IMPDH, mycophenolic acid (MPA), is used as an immunosuppressant drug to prevent rejection in organ transplantation by inhibiting proliferation of T cells and B cells. Several features of *IMPDH* make it an attractive anti-microbial drug target^12^. While microbes can take up purine bases in the environment by salvage pathways, concentrations of purines in human plasma and other extracellular fluids are insufficient for microbial survival^13^, suggesting that *IMPDH* might be essential for virulence *in vivo*. Furthermore, structural differences between microbial and human IMPDHs suggest that an inhibitor that is specific to the fungal enzyme could be attained. Among fungal species, deletion of *IMPDH* results in avirulence of *Cryptococcus neoformans* ^14^. MPA inhibited *in vitro* growth of *Candida albican*, whereas overexpression of IMPDH confers resistance to MPA^15^, suggesting that enzymatic activity of IMPDH is critical for growth of *C. albicans*. In addition, another key enzyme in the *de novo* guanine biosynthesis, guanosine monophosphate (GMP) synthase that catalyzes XMP to GMP, has been shown essential for fungal viability and virulence in *A. fumigatus* and *C. albicans* ^16,17^. However, it remains to be ascertained if *IMPD*H is essential for virulence of *A. fumigatus*.

To determine whether *IMPDH* is essential for *A. fumigatus* viability, we created the *AfIMPDHΔ* and the *Tet-Off* promoter driven *AfIMPDH* strains (*P*_*Tet-Off*_ *-AfIMPDH*) by homologous recombination. As expected, *AfIMPDHΔ* failed to grow in the absence of guanine, whereas addition of guanine restored its growth (Fig. 1c). Addition of doxycycline to the guanine-free media, ranging from 1 to 50 μg/mL, greatly inhibited, but did not completely abolish the growth of *P*_*Tet-Off*_ *-AfIMPDH* strain. In addition, the growth of *P*_*Tet-Off*_ *-AfIMPDH* strain was restored by supplementing guanine in the media (Fig. 1b, data not shown).

### *IMPDH* is not essential for virulence of *A. fumigatus in vivo*

**Figure 2:**
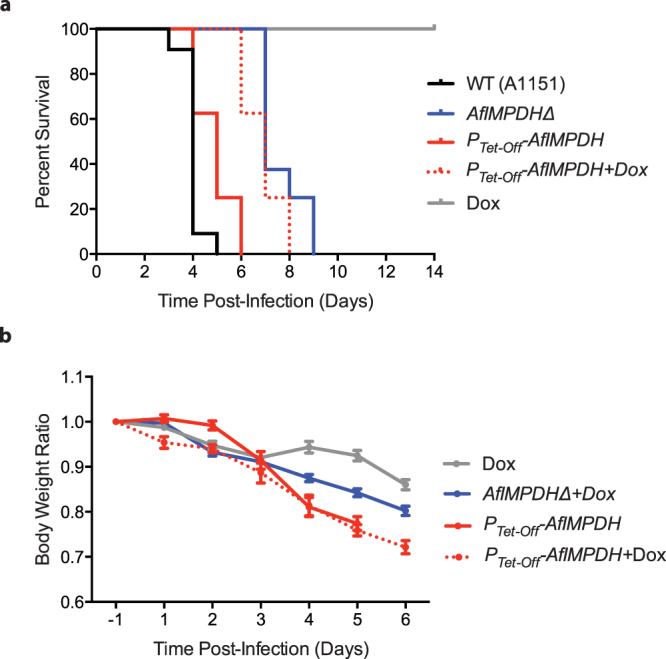
Deletion of *AfIMPDH* attenuates but does not completely abolish virulence of *A. fumigatus*. (**a**) Survival curves of mice with invasive pulmonary aspergillosis (Kaplan-Meier plot). Mice were immunocompromised by injection of cyclophosphamide intraperitoneally and cortisone acetate subcutaneously before infection. Freshly prepared conidia (5 × 10^4^) of wild type (A1151), *AfIMPDHΔ*, and *P*_*Tet-Off*_ *-AfIMPDH* strains were intranasally inoculated under anesthesia. Doxycycline was administered at day −4 (4 days before infection) to turn off expression of *AfIMPDH*. Each group includes more than eight mice. (**b**) Body weight change after infection. The body weights of each day after infection are compared to the body weights at day −1. Averages and the standard errors of the mean are presented.

We next tested whether loss of *AfIMPDH* affects virulence of the pathogen using an invasive pulmonary aspergillosis mouse model^18^. Seven-week-old female BALB/c mice were immunosuppressed and then intranasally infected with 5 × 10^4^ conidia of wild type, *AfIMPDHΔ* or *P*_*Tet-Off*_ *-AfIMPDH* strains. Mortality and body weight were monitored daily. Surprisingly, deletion of *AfIMPDH* attenuated, but did not completely abolish virulence of *A. fumigatus*. While mice infected with the wild type conidia showed the median survival time of 4 days, mice infected with the *AfIMPDHΔ* conidia showed the median survival time of 7 days (Fig. 2a) (p < 0.0001).

We further tested whether doxycycline treatment could recapitulate the attenuated virulence of the *P*_*Tet-Off*_ *-AfIMPDH* strain *in vivo*. Similar to wild type, infection with *P*_*Tet-Off*_ *-AfIMPDH* conidia had the median survival time of 5 days. To repress the expression of *AfIMPDH*, doxycycline was administered by oral gavage twice daily to mice from 4 days prior to infection for a total 10 days. We chose this dosage regimen because it has been shown to effectively induce Tet-inducible gene expression in a mouse subcutaneous xenograft model^19^. At this given dose, the serum steady state doxycycline level could reach to about 10 μg/mL in mice^20^, which is 10 fold the *in vitro* doxycycline concentration (1 μg/mL). We observed that mice given doxycycline survived 2 days longer than mice without doxycycline treatment (Fig. 2a) (p = 0.0008). Doxycycline-treated mice had the same median survival time (7 days) as the mice infected with the *AfIMPDHΔ* conidia, suggesting that the expression of *AfIMPDH* is effectively repressed. We also note that doxycycline treatment caused body weight reduction (Fig. 2b), as observed previously^19^.

Taken together, our data show that our Tet-off expression system can repress expression of *AfIMPDH in vivo*. Additionally, our results demonstrate that *AfIMPDH* is not essential for virulence and is therefore not a good drug target for *A. fumigatus*. A previous study showed that GMP synthase, an enzyme downstream of *AfIMPDH* in the *de novo* guanine biosynthesis, is essential for virulence^16^. We noted that a murine model of systemic aspergillosis was used in the study. Since the growth of *A. fumigatus* mutants defective in *de novo* guanine biosynthesis is solely dependent on concentration of guanine in the tissue, we postulate that difference could be due to the fact that extracellular concentration of purines in lung tissue is relatively higher than that in blood and kidney and may be sufficient to bypass the lack of *AfIMPDH*.

### *sidA* is conditionally essential *in vitro* and essential *in vivo* for *A. fumigatus*

**Figure 3:**
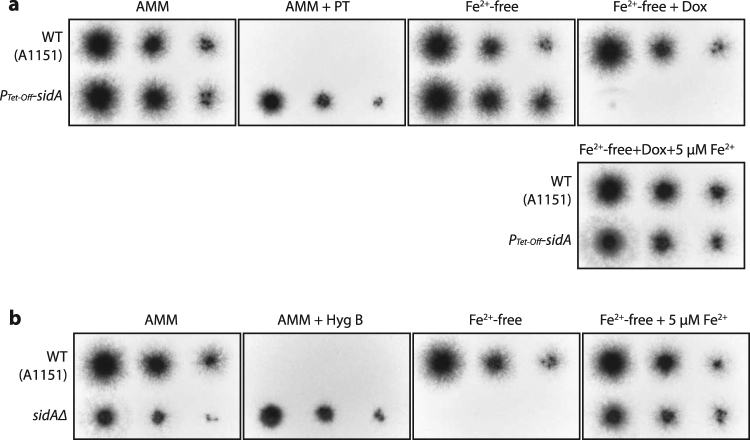
Growth of the *sidAΔ and P*_*Tet-Off*_ *-sidA* mutants *in vitro*. (**a**) Doxycycline-dependent growth of the *P*_*Tet-Off*_ *-sidA* strain. (**b**) Growth of the *sidA*Δ strain. Conidia of the wild-type and the mutant strains were spotted and grown as described in Fig. 1.

We next tested *sidA*, a gene that is known to be essential for *A. fumigatus* virulence^21,22^, to further validate our *Tet-Off* system. Iron is essential for growth of *A. fumigatus* but is poorly available in animal hosts. *A. fumigatus* depends on siderophore, an iron-specific chelator, to obtain iron *in vivo*. The *sidA* gene encodes L-ornithine N^5^-oxygenase that catalyzes the first committed step in hydroxamate siderophore biosynthesis^23^. Deletion of *sidA* leads to growth defect in iron-limited media and complete avirulence in murine models of invasive aspergillosis^21,22^. We created *sidA* null and *Tet-Off* promoter driven *sidA* (*P*_*Tet-Off*_ *-sidA*) mutants and compared their growth. In the absence of iron, growth of *sidAΔ* was completely abolished (Fig. 3b). Likewise the *P*_*Tet-Off*_ *-sidA* mutant did not grow in the presence of doxycycline at the concentration of 1 μg/mL (Fig. 3a), indicating that *sidA* expression was effectively repressed by doxycycline. Moreover, addition of ferrous ion (Fe^2+^) restored the growth of *sidA* null mutants (Fig. 3a). A previously reported Tet-Off gene expression system for *A. fumigatus* inhibited gene expression significantly *in vitro* in the presence of doxycycline at a concentration of 50 μg/mL^10^. In comparison, we found that our Tet-Off system greatly inhibited gene expression of *AfIMPDH* and *sidA* at 1 μg/mL concentration of doxycycline and increased doxycycline concentration did not enhance the inhibition. Future studies will be needed for direct comparison of these two Tet-Off systems.

**Figure 4:**
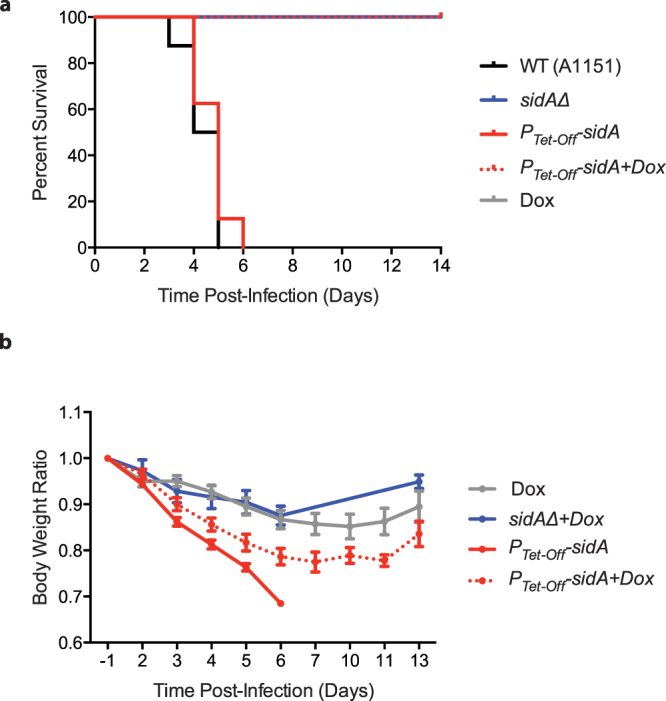
Depletion of *sidA* abolishes virulence of *A. fumigatus*. (**a**) Survival curves for mice with invasive pulmonary aspergillosis (Kaplan-Meier plot). Mice were immunocompromised as previously described and were infected with conidia of wild type (A1151), *sidAΔ*, and *P*_*Tet-Off*_ *-sidA* strains. Doxycycline was administered at day −4 to turn off expression of *sidA*. Each group includes more than eight mice. (**b**) Body weight change after infection. The body weights of each day were monitored and presented as described in Fig. 2.

We next tested virulence of the *sidAΔ* and *P*_*Tet-Off*_ *-sidA* mutants. In contrast to the wild type conidia, the *sidAΔ* conidia were avirulent (p = 0.0001), consistent with previous reports^21,22^. The *P*_*Tet-Off*_ *-sidA* conidia demonstrated similar virulence to the wild type in the absence of doxycycline, while the conidia were completely avirulent in the mice administered with doxycycline (Fig. 4a) (p < 0.0001), validating the effectiveness of the Tet-Off gene expression system for controlling gene expression *in vivo*. We again observed that doxycycline treatment caused body weight reduction (Fig. 4b).

### Control of *sidA* expression in a time-dependent manner *in vivo*

**Figure 5:**
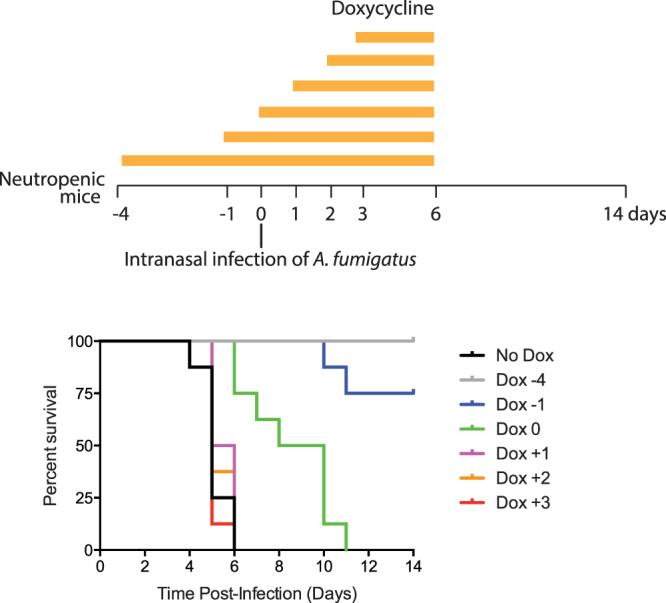
Control of *sidA* expression in host animals in a time-dependent manner. Mice were immunocompromised and were infected with conidia of the *P*_*Tet-Off*_ *-sidA* strain. Doxycycline was administered at day −4, −1, 0, +1, + 2, + 3 to regulate expression of *sidA*. The control group (No Dox) represents the mice without doxycycline treatment.

We sought to test whether the Tet-Off system can regulate expression of a target gene in a time-dependent manner *in vivo*. Previously, we administered doxycycline 4 days prior to infection for continuously 10 days and observed 100% survival of mice (Figs 4a and 5). The critical factor to turn off the expression of *sidA* is the concentration of doxycycline in mouse lung tissue. It has been reported that when given to human patients orally doxycycline reached at relatively stable concentration in bronchial secretions about 24 hours after the first dose^24^. Therefore we administered the first dose of doxycycline to mice at −1, 0, 1, 2 and 3 days after infection to mimic the dosing regimen that patients receive with antifungal therapy in the clinic at 0, 1, 2, 3 and 4 days after infection, respectively. We observed that administering doxycycline at day −1 is very effective, and rescued 75% of mice (Fig. 5) (p < 0.0001); and administering doxycycline at day 0 also significantly prolonged the medial survival time from 5 days of control to 9 days (p = 0.0005). In contrast, administering doxycycline after day 1 (day 1, 2 and 3) showed very little effect on the survival rate of mice (Fig. 5). These results demonstrate that the timing of doxycycline administration dramatically affects survival rates.

Previous animal studies reported that delayed antifungal therapy is ineffective for invasive pulmonary aspergillosis due to an extensive hyphal invasion in the first 48 hours post infection^25,26^. Unfortunately delayed antifungal treatment can often be the case in the clinic. Therefore it is of great interest to develop antifungal therapy that is effective after conidia enter hyphal growth phase. Our Tet-Off system provides a potential way to test targets for such therapy. Moreover, molecular mechanism underlying conidia transition from dormancy to germination is still largely unknown. Transcriptome and proteome studies revealed that over one third of *Aspergillus* genome is differentially expressed when conidia enter germination *in vitro* ^27–31^. This Tet-Off system can be used to verify if a given gene is required for germination and/or hyphal growth *in vivo*.

## Methods

### Strains and Media

The *Aspergillus fumigatus* strains used in this study are listed in Table 1. Conidia were collected from cultures grown on potato dextrose agar (Sigma-Aldrich) at 37 °C. The growth of the wild type and mutant conidia were tested on *Aspergillus* Minimal Media (AMM) plates supplemented with 200 μg/mL hygromycin B (HygB), 0.2 μg/mL pyrithiamine (PT), 1 μg/mL doxycycline (Dox), FeSO_4_ (Fe^2+^), or 500 μM guanine at 37 °C.

**Table 1:** Strains used in this study.

Strain	Genotype	Reference
A1151	*AfKU80Δ::pyrG*	Fungal Genetics Stock Center
CPA24	*AfKU80Δ::pyrG sidAΔ::HygB* ^*R*^	This study
CPA34	*AfKU80Δ::pyrG P*_*Tet-Off*_ *-AfIMPDH::PtrA* ^*R*^	This study
CPA41	*AfKU80Δ::pyrG AfIMPDHΔ::HygB* ^*R*^	This study
CPA44	*AfKU80Δ::pyrG P*_*Tet-Off*_ *-sidA::PtrA* ^*R*^	This study

### Plasmid construction

The Tet-Off gene expression cassette was constructed based on previously published Tet-On gene expression cassette in pCH008^7^. The reverse tetracycline transactivator *rtTA2* ^*S*^ *-M2* was replaced with the tetracycline transactivator tTA-Advanced (*tTA2* ^*S*^) (Clontech) using Gibson Assembly strategy (NEB). The final Tet-Off cassette is composed of *PtrA* resistance cassette, promoter of *tpiA*, *tTA2* ^*S*^, terminator of *cgrA*, *tetO* promoter and a minimal promoter.

### Strain construction

Using a method based on homologous recombination described previously^11^, the target genes were deleted with a hygromycin B resistance cassette or the promoters of the target genes were replaced with the Tet-Off gene expression cassette in a non-homologous end-joining-deficient strain (A1151, Fungal Genetics Stock Center). First, approximately 1 kb upstream and downstream of the targeted chromosome region were amplified by PCR from genomic DNA of A1151. These two DNA fragments were assembled with the hygromycin B cassette or Tet-Off gene expression cassette in pUC19 using NEB Gibson assembly kit. Second, the resultant deletion cassette or Tet-Off cassette flanked with the targeted gene fragments were PCR amplified and purified. *A. fumigatus* was transformed by electroporation as described previously^32^. The transformants were selected on AMM supplemented with HygB (for deletion) or PT (for Tet-Off promoter replacement). The mutant strains were confirmed by colony PCR.

### *A. fumigatus* virulence studies

Seven-week-old female BALB/c mice weighing 18–20 g (The Jackson Laboratory) were used for the invasive pulmonary aspergillosis model^18^. Immunosuppression was achieved by injection of cyclophosphamide (Baxter Healthcare) intraperitoneally 4 days at 150 mg/kg and 1 day at 100 mg/kg before inoculation and injection of cortisone acetate at 250 mg/kg (Sigma) subcutaneously 1 day before inoculation. On inoculation day, 40 μL of *A. fumigatus* conidial suspension containing 5 × 10^4^ freshly collected conidia in sterile PBS + 0.02% Tween 20 were intranasally inoculated under anesthesia. Additional doses of cyclophosphamide (100 mg/kg) were given on days 2 and 6 after inoculation to maintain neutropenia. Mortality was assessed daily until day 14 after inoculation. Animals displaying signs of morbidity were euthanized, and their death was recorded as occurring 12 hours later^19^. 200 μL of 10 mg/mL doxycycline were treated twice a day via *gastric lavage*. Statistical analyses for comparison of survival rate between WT and knockout, or between *P*_*Tet-Off*_ *-target gene* with doxycycline and without doxycycline were conducted using Prism 6 and p values from the Log-rank (Mantel-Cox) test were presented.
